# De novo evolution of transmissible tumours in hydra

**DOI:** 10.1098/rspb.2024.1636

**Published:** 2024-09-18

**Authors:** Sophie Tissot, Jordan Meliani, Justine Boutry, Lionel Brazier, Jácint Tökölyi, Benjamin Roche, Beata Ujvari, Aurora M. Nedelcu, Frédéric Thomas, Antoine M. Dujon

**Affiliations:** ^1^ CREEC/MIVEGEC, Université de Montpellier, CNRS, IRD, Montpellier, France; ^2^ Department of Evolutionary Zoology, MTA-DE “Momentum” Ecology, Evolution and Developmental Biology Research Group, University of Debrecen, Debrecen 4032, Hungary; ^3^ Departamento de Etología, Fauna Silvestre y Animales de Laboratorio, Facultad de Medicina Veterinaria y Zootecnia, Universidad Nacional Autónoma de México (UNAM), Ciudad de México, Mexico; ^4^ School of Life and Environmental Sciences, Deakin University, Waurn Ponds, Victoria, Australia; ^5^ Department of Biology, University of New Brunswick, Fredericton, New Brunswick, Canada

**Keywords:** neoplasm, transmissible cancer cells, experimental evolution, cnidarian

## Abstract

While most cancers are not transmissible, there are rare cases where cancer cells can spread between individuals and even across species, leading to epidemics. Despite their significance, the origins of such cancers remain elusive due to late detection in host populations. Using *Hydra oligactis*, which exhibits spontaneous tumour development that in some strains became vertically transmitted, this study presents the first experimental observation of the evolution of a transmissible tumour. Specifically, we assessed the initial vertical transmission rate of spontaneous tumours and explored the potential for optimizing this rate through artificial selection. One of the hydra strains, which evolved transmissible tumours over five generations, was characterized by analysis of cell type and bacteriome, and assessment of life-history traits. Our findings indicate that tumour transmission can be immediate for some strains and can be enhanced by selection. The resulting tumours are characterized by overproliferation of large interstitial stem cells and are not associated with a specific bacteriome. Furthermore, despite only five generations of transmission, these tumours induced notable alterations in host life-history traits, hinting at a compensatory response. This work, therefore, makes the first contribution to understanding the conditions of transmissible cancer emergence and their short-term consequences for the host.

## 1. Introduction

The evolution of animal multi-cellularity at the end of the Precambrian was accompanied by the ability to regulate cell proliferation in a spatio-temporal context. However, mutations in these regulatory systems can occur, leading to uncontrolled proliferation that can result in neoplasms (tumours) that, in some cases, transform into invasive and deadly cancers [1,2]. Even though malignant progressions can and do occur in the majority of multi-cellular organisms, only a few cases of transmissible cancers have been reported (although their number could be underestimated, see [3,4]). Currently, 14 transmissible cancers are known in the wild: two affecting the Tasmanian devil [5], one observed in the Canidae [6] and 11 identified in bivalves (in some of the latter cases, they also exhibit the capacity to cross the species barrier) [7–11]. The transmissibility of these cancer cells aligns their evolutionary dynamics more closely with those of emerging pathogens, fostering long-term coevolution with their host. For example, approximately 40 years after its manifestation [12], the transmissible cancer line associated with the devil facial tumour disease in Tasmanian devils seems to evolve into an obligatory parasite that genuinely coevolves with its host [13].

Transmissible tumour cells, in addition to expressing the hallmarks of cancer cells [14], have to match a series of exact conditions (i.e. a ‘perfect storm’) to emerge and spread as an epidemic. According to Ujvari *et al*. [15], the perfect storm theory considers a first barrier made of at least four key factors, namely: (i) the release of tumour cells from the impacted host, (ii) the persistence of tumour cells during transit between hosts, (iii) a conducive environment supporting invasion, and (iv) the ability to adapt to new environments while avoiding immune responses in the foreign host. In 2022, Tissot *et al*. [16] added an additional hurdle to the emergence of transmissible cancer, beyond acquiring transmissibility itself: the ability for dissemination within host populations, at least until a prevalence threshold is reached, triggering the epidemic. The key parameters influencing the crossing of this second barrier, dissemination, pertain to abiotic or biotic variables that act directly on the cancer cells (e.g. water current in bivalves [17]) and/or on the infected hosts (e.g. predation, parasitism, [15,17–20]), as well as long-term genetic factors (e.g. accumulation of deleterious mutations [21,22]), potentially halting the outbreak of an epidemic even if the first transmissibility barrier has been breached. The many conditions required to create a perfect storm could explain the rarity of transmissible cancers [16]. Furthermore, a significant limitation to our understanding of the conditions leading to the emergence of transmissible cancers is that their presence is typically observed only after they have spread widely within host populations, i.e. relatively long after their initial appearance (e.g. [12]).

The freshwater cnidarian *Hydra oligactis* has been observed to spontaneously develop tumours under laboratory conditions, particularly in response to extensive feeding [21,23]. In a notable case that occurred 15 years ago in Thomas Bosch’s laboratory in Germany, a hydra developed a tumour, consisting of overproliferation of large interstitial stem cells, capable of being transmitted vertically through the asexual reproduction of its host, by budding. The isolation and culture of this polyp enabled the establishment of a tumoural hydra line, referred to as the St Petersburg strain [24]. As the bud detaches from the tumoural hydra, it undergoes an initial pre-pathological phase, during which the tumours have not yet developed to the point of being detectable externally. However, after approximately four or five weeks, tumours become visible, marking the transition into the pathological phase [24]. Subsequent research revealed that some of these transmissible tumours were linked to a specific bacteriome, notably involving the co-occurrence of spirochetes and *Pseudomonas* [25]. Additionally, Boutry *et al*. [26] recently demonstrated that some wild *H. oligactis*, when brought back to the laboratory, develop spontaneous tumours at relatively high frequencies (i.e. up to 30%, see [27]), depending on the population of origin. However, it remains unclear to what extent these spontaneous tumours are already transmissible, and/or if this trait can be acquired over time. In this context, the hydra-tumour model appears to be an excellent model for attempting to explore, for the first time, the evolution of transmissible tumours.

In this study, employing *H. oligactis*, we observed for the first time, the experimental evolution of a transmissible tumour. Our aim was to assess the initial transmission rate of these tumours and explore the potential for optimizing this rate through selection. One of the selected strains was then examined through cell type analysis, and its bacteriome as well as life-history traits were explored.

## 2. Material and methods

### (a) Sampling of wild hydra and tumour induction

**Figure 1.**
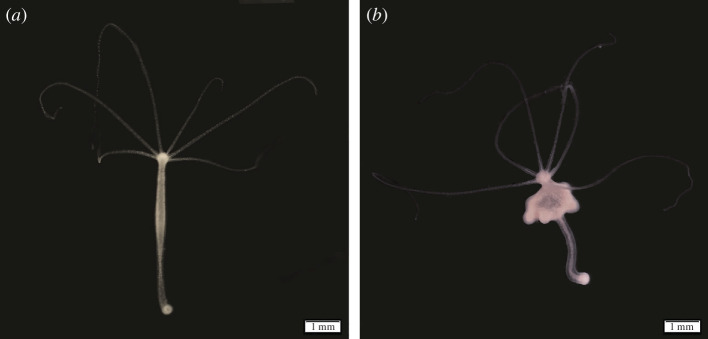
Phenotype of tumour-free and tumoural hydras from the laboratory population established with individuals sampled from Montaud lake. (*a*) Tumour-free hydra from the control population: the body is long and thin. (*b*) Hydra from the tumoural strain presenting numerous masses thickening the body. A trinocular magnifier was used to take the pictures, scale bar: 1 mm.

The hydras used in this study were collected from the Montaud lake in France (43°44’52” N; 3°59’23” E) on 2 May 2022. Fifty hydras were individually maintained at 18°C in cell culture plates (12-well plates, 1.5 ml per well, Thermo Scientific) filled with Volvic© water under a photoperiod of 12 h. A previous study on hydra suggests that high dietary availability may favour the development of tumour cells once they have appeared, by stimulating cell proliferation and providing the energetic resources necessary for their growth [27]. Then, to ensure a high rate of tumour development [27], budding [28] and thus the chances of tumour transmission, some of the polyps were fed ad libitum five times a week with nauplii of *Artemia salina*, obtained as described in Boutry *et al*. [29]. The wells were cleaned 8 h after feeding by removing the leftover artemia and refilled with Volvic© water. The development of tumour in this initial generation of hydras and in their descendants was characterized with the help of the scale used by Tissot *et al*. [27]. Hydras were considered as tumoural when they showed at least one medium deformation of their body (figure 1). An additional 100 polyps, placed in mass culture, were fed ad libitum only three times a week to restrict the occurrence of spontaneous tumours, consequently serving as the initial (F0) control population of tumour-free hydras (CTRL).

### (b) Selection for tumour transmissibility

**Figure 2.**
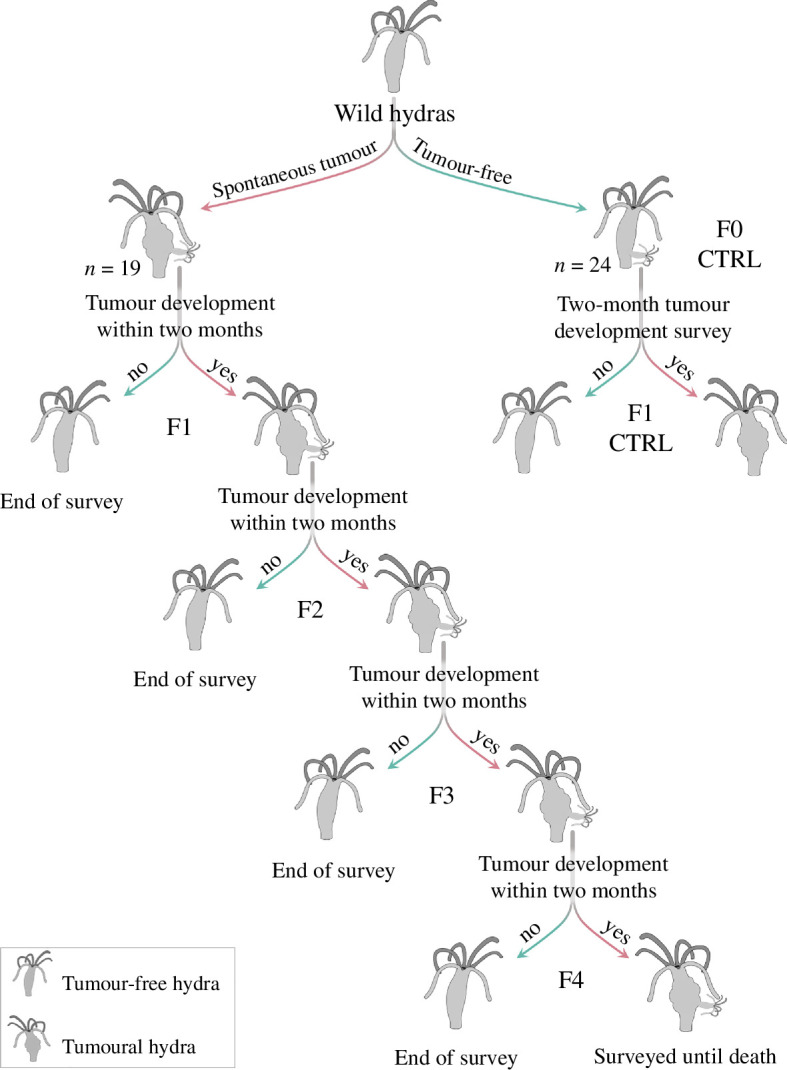
Illustration of the protocol for selection on tumour transmissibility.

To estimate initial tumour transmission rate, we isolated 19 individuals (F0) from the hydras fed five times a week as soon as they have developed spontaneous tumours, and collected all the buds that they subsequently produced until their death. These buds (F1) were placed individually under the same conditions as their parents, and their health status (i.e. development of tumour or not) over time was monitored. When a tumour develops in the F1 cohort, it may be due to transmission from the F0 parents, or to the spontaneous development of a tumour in an F1 individual. In an attempt to control for the rate of spontaneous tumour emergence, we simultaneously isolated buds (F1 CTRL) from the initial population of tumour-free hydras (F0 CTRL) and placed them individually under the same rearing conditions for two months, with five feedings per week. At the end of this period, their health status (i.e. tumour-free, tumoural or dead) was recorded. In buds from F0 tumoural parents, if tumours appeared within two months, the date of tumour appearance was recorded. Then, all buds produced after that date until the individual’s death were isolated and surveyed in the same manner as their parents. If no tumours appeared within two months, the survey was stopped, and the health status was recorded as ‘tumour-free’. If an individual died during the experimentation, the associated date was also recorded, and the status was marked as ‘dead’. This process was repeated during eight months in order to obtain a total of four generations and was then stopped for logistic reasons. Figure 2 summarizes the protocol used to select for tumour transmissibility from hydras with spontaneous tumours.

### (c) Characterization of a selected hydra strain with transmissible tumours

#### (i) Life-history traits

**Figure 3.**
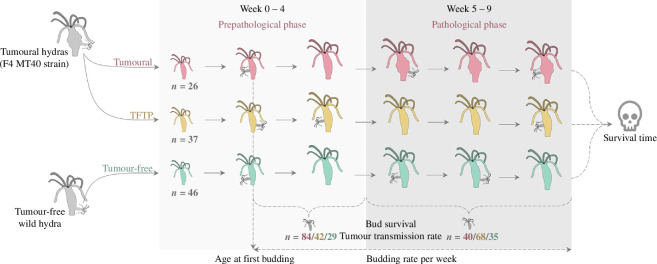
Graphical summary of the protocol for life-history trait measurements.

The fifth generation of a tumoural hydra strain, namely MT40, obtained from the selection experiment described above, was used to quantify its life-history traits. We used this strain because it had the highest budding rate, allowing us to obtain enough individuals to carry out this type of analysis. The tumour-free polyps from the same initial population were used as controls. To estimate the asexual reproductive effort, we monitored 46 buds from tumour-free hydras and 63 from the MT40 tumoural strain over six months. Of the 63 hydras derived from the tumoural strain, 26 have developed tumours and were identified as tumoural, while the 37 that have not developed tumours will henceforth be referred to as TFTP (tumour-free hydra from tumoural parent). Figure 3 summarizes the protocol used to measure life-history traits on the strain of interest and its control. This analysis involved tracking the age at first budding, the weekly bud count for nine weeks, bud survival at two months, tumour transmission rate (i.e. the number of infected buds out of the total bud count) and the survival time (in days). To measure bud survival and tumour transmission rate, firstly during weeks 0–4, we isolated 84 buds from tumoural hydras during their pre-pathological phase (i.e. before tumour appearance, see §1), 42 buds from TFTP and 29 buds from tumour-free hydras as control. Then, from the same polyps, during weeks 5–9 corresponding to the pathological phase for tumoural hydras (i.e. after tumour appearance), we isolated 40 buds from tumoural polyps, 68 from TFTP and 35 from tumour-free hydras as control. All the buds isolated in the pre-pathological and in the pathological phase were surveyed for two months, at the end of which their status was evaluated (i.e. tumoural, tumour-free or dead).

#### (ii) Bacteriome and cell type

To characterize the aetiology of the tumours, we conducted a bacteriome and cell type analysis. This aimed to determine: (i) whether they are associated with a specific bacteriome, as observed in the transmissible hydra tumours described by Domazet-Lošo *et al.* [24] and (ii) whether the cell type forming the tumour is similar to that reported by Boutry *et al.* [26] in the same hydra population.

For the bacteriome analysis, we utilized five tumour-free hydras from the control population, five tumoural hydras and five TFTP. The water from the wells of each hydra status was also sampled to control that the bacteriome of the water did not influence those of the hydras. They were first washed in three baths of sterile water (autoclaved MilliQ water) before being placed at −20°C for storage. DNA was extracted with the Qiagen Blood & Tissue Kit according to the manufacturer’s instructions, and DNA concentration was measured using a Qubit 2.0 fluorometer. The primers 5’GTGCCAGCMGCCGCGGTAA and 3’GGATTAGAWACCCBDGTAGTCC were used to target the V4 region of the 16S gene during the polymerase chain reaction (PCR). The products were then sent to the genomic platform (Genseq, Montpellier University) for Illumina sequencing.

For the cell type analysis, two individuals from each status, namely tumour-free (as control), tumoural and TFTP were macerated together and 100 µl of the solution were spread on gelatin-coated microscope slides according to the procedure detailed in David [30]. Once dry, the slides were observed by phase contrast under a microscope with a 40× objective, and the number of epithelial, small interstitial stem cells and large interstitial stem cells were counted.

### (d) Data analysis

The analyses presented here were performed with the R software (v. 4.2.2) [31] and the graphical representations were realized with the ‘ggplot2’ package [32]. The sequencing data for the bacteriome were checked for their quality and processed via the FROGS analysis pipeline [33] developed by the GenoToul genomic platform in the Galaxy interface.

#### (i) Selection for tumour transmissibility

To analyse: (i) the proportion of tumoural hydras in the first generation (F1) according to the parental status (i.e. tumoural or tumour-free) and (ii) the tumour transmission rate according to generation and bud order (i.e. the ratio of bud rank on total bud number per parent), we used generalized linear mixed-effects models (GLMMs) from the package ‘glmmTMB’ [34], as the response variables here were non-normally distributed (i.e. binary or count data). For the proportion of hydras developing tumours in F1, a random intercept effect of the birth date (i.e. date of polyp detachment from parental hydra) was added to consider the possible temporal variability, and a binomial distribution was used. Concerning tumour transmission rate, a random intercept effect of the founder individual (i.e. F0 strain) was added to consider possible variability between strains, and a binomial distribution was applied. In this analysis, buds without siblings were excluded (constituting 3 cases out of 280) to specifically investigate the impact of bud order on tumoural transmission. According to Zuur *et al*. [35], model selection was made first on the random effect and then on the fixed effect, based on the weight of the corrected Akaike information criterion (AICc) obtained with the package ‘MuMIn’ [36]. Then, the fitting of the model obtained was checked with ‘DHARMa’ package [37] which performs a Kolmogorov–Smirnov, outlier and overdispersion test on model residuals. The detail of the variable types (i.e. in this case, continuous, date or factor variables) as well as all the models constructed and the AICc weight associated is presented in electronic supplementary material, table S1.

#### (ii) Life-history traits

To analyse: (i) first bud date according to status, (ii) budding rate according to status and phase (i.e. from the first bud date to the week 4 and weeks 5–9, corresponding to the pre-pathological and pathological phases of tumoural hydras, respectively), (iii) bud survival according to the status and the phase, and (iv) tumour transmission rate according to status and phase, we used GLMMs. Concerning the age at first budding and the budding rate analysis, a random intercept effect of birth date was added to control variability linked, respectively, to different batches, and only for budding rate the random effect of the individual was also added to consider the repeated measurements on the same individuals. A random effect of the parent’s identity was added to analyse bud survival and tumour transmission rate, as buds collected in both phases come from the same parents. For the analysis of the first two traits, a negative binomial distribution was used as the age at first budding counts the number of failures (i.e. days) before the success (i.e. the first bud production) and the budding rate is too low to use a Poisson distribution (i.e. the variable is underdispersed). For the last two variables, a binomial distribution was used as they are both binary. The model selection and validation are identical to the previous section. With regard to the age at first budding, an outlier was removed from the dataset (58 days versus an average of 17) after being detected by the outlier test during verification of the established model, which prevented a good fit. Concerning the analysis of bud survival and tumour transmission rate, as only one control individual developed a spontaneous tumour, it was removed from the dataset in order to test the presence of an interaction between status and phase. The analysis of tumour transmission rate was performed only on buds from tumoural hydras and TFTP, as no tumour appearance was observed on buds from control hydras.

For the analysis of the survival time according to the status, survival regressions were used with the exponential distribution, as the instantaneous risk was constant (verification by plotting the survival curve of individuals). The selection model was also based on the AICc weight. The details of variable forms, as well as all the models tested and their AICc weights, are presented in electronic supplementary material, table S2.

#### (iii) Cell type and bacteriome

Only the ratio between interstitial stem cells and epithelial cells has been calculated on the data collected after maceration (see [26]). The bacterial feature table obtained from the FROGS pipeline was used to compare samples with each other. The taxonomic affiliation of each operational taxonomic unit (OTU) used RDPtools with the 16S SILVA 138.1 reference database. As a first step, we compared bacterial microbiota composition of polyp samples with their respective water samples, to evaluate whether polyp samples differentiate from their environment, as expected if our 16S profiling identified polyp-associated bacteria rather than environmental contaminants. Next, polyps from different status groups (tumoural, TFTP and tumour-free) were compared in terms of alpha and beta diversity at the bacterial species (i.e. feature) level. For alpha diversity, we calculated the chao1 index as a proxy for bacterial species richness and used Kruskal–Wallis test to compare treatment groups. For beta diversity, we calculated distance between samples using the Bray–Curtis index (which takes bacterial abundance into account) and the Jaccard index (which takes only presence–absence data into account). These distance matrices were subsequently compared using Adonis with *n* = 999 permutations and pairwise Adonis to test the statistical null hypothesis that the bacteriome composition of treatment groups is not differentiated. All these analyses were subsequently repeated at the bacterial genus and family level to make sure that results are robust to taxonomic resolution. Abundance data for the most common bacteria were plotted at the order level in the form of taxonomy barplots available in electronic supplementary material, figures S2–S4. Bacteriome analyses were performed with the microbiome [38], vegan [39] and pairwiseAdonis [40] R packages.

## 3. Results

### (a) Selection for tumour transmissibility

**Figure 4.**
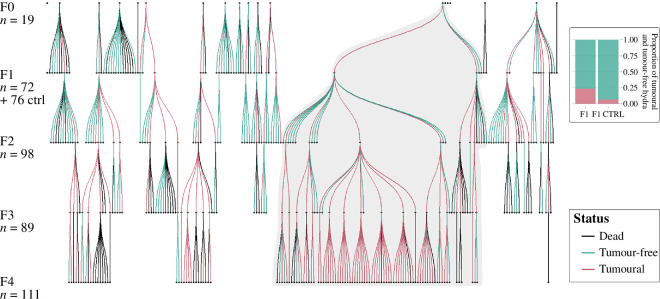
Summary of tumour transmission selection results and the proportion of hydras developing tumours in F1 and F1 CTRL. The MT40 strain, used for life-history trait analysis in the second part, is shaded.

To assess the conditions associated with the emergence and evolution of transmissible tumours in *H. oligactis*, we investigated the presence of tumours in the offspring (F1) developed from buds of wild individuals that had developed spontaneous tumours (F0). For those that had developed tumours, their offspring were also investigated over three additional generations. The results of this experimental selection are summarized in figure 4. To control for the rate of spontaneous tumour appearance, buds from tumour-free hydra from the same wild population were surveyed in the same way.

#### (i) Prevalence of tumours in the first generation

In the first generation, F1 hydras were approximately four times more likely to develop tumours when they came from tumoural F0 parents than from tumour-free parents (figure 4; GLMM, Odds Ratio (OR) = 4.27, s.e. = 2.30, *p*‐value = 0.007). In the tumour-free population, approximately 7% (i.e. 5 out of 74) of individuals developed tumours, while in the tumoural strain, approximately 24% (i.e. 17 out of 72) developed tumours.

#### (ii) Estimation of transmission rate over generations

**Figure 5.**
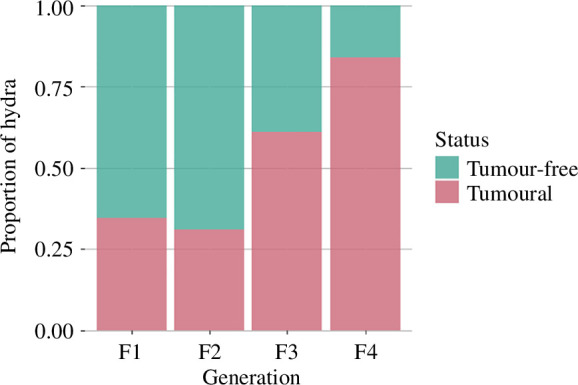
Proportion of hydras developing tumours according to generation.

Concerning the tumour transmission rate, the model selected included the additive effect between generations and bud order, with a random effect of the founder individual. The tumour transmission rate more than doubled over the course of the experiment, from 35% in the first generation to 84% in the fourth (figure 5; GLMM, *generation effect*, OR = 2.13, s.e. = 0.37, *p*‐value < 0.001). The order of the bud formation has no significant effect on the transmission rate. Thus, the tumour transmission rate responded positively to artificial selection, but its occurrence among buds seemed to be random within an individual line.

### (b) Life-history traits

Using hydras from the tumoural strain MT40 (established during the previous tumour transmission selection), and tumour-free wild hydras as control, we analysed: (i) age at first budding, (ii) asexual reproduction effort, (iii) bud survival, (iv) transmission rate, and (v) survival time, according to the status (i.e. tumour-free, tumoural and TFTP) and the phase (i.e. weeks 0–4, and weeks 5–9 matching for tumoural hydra to the pre-pathological and pathological phases) when relevant.

The status of the hydra had no effect on the age at first budding, as indicated by the model selection. On average, the first budding occurred at approximately 17 days regardless of whether the hydra was tumoural or not.

**Figure 6.**
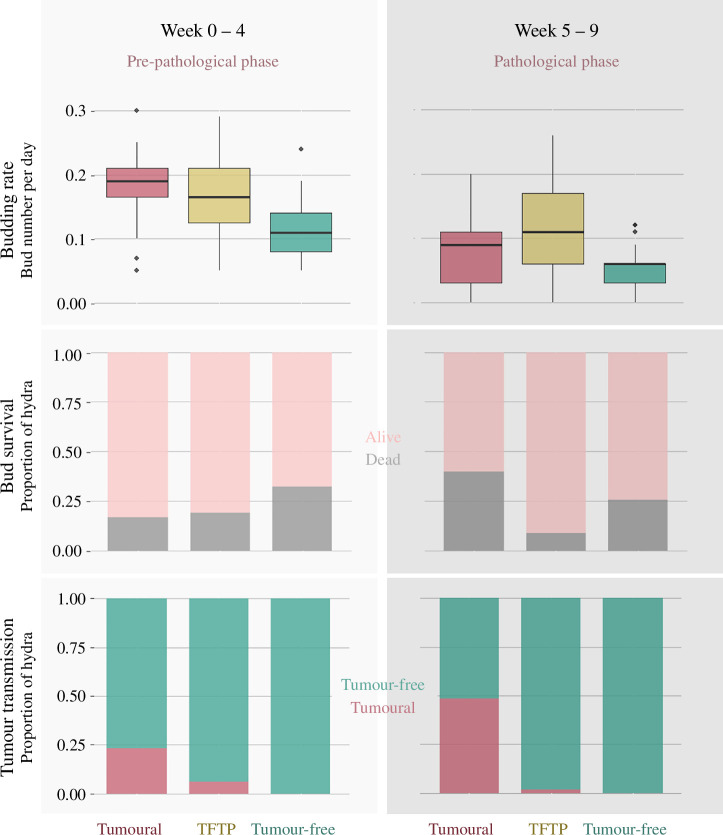
Budding rate, bud survival within two months and tumour transmission rate according to hydra status for each phase.

The budding rate was explained by the interaction between the status of hydras and the phase with a random effect of the individual. Precisely, before five weeks, tumour-free hydras produced approximately 30% less buds than tumoural hydras and TFTP (figure 6; GLMM, *status effect*, Incidence Rate Ratio (IRR) = 0.68, s.e. = 0.09, *p*‐value = 0.002). However, tumoural hydras were the only ones for which a decrease in the budding rate was observed after the fifth week, which corresponds to the start of the pathological phase for these hydras (figure 6; GLMM, *interaction effect of status and phase*, IRR = 0.61, s.e. = 0.13, *p*‐value = 0.024). The budding rate of tumoural hydra in pathological phase was reduced by approximately 40% compared with the pre-pathological phase.

For bud survival, the interaction effect between the phase in which the parents were at the time of bud production and the status were retained, as well as the random effect of parent identity. Buds of the three statuses showed a similar mortality risk, approximately 25%, when they are produced during weeks 0–4. Only buds from tumoural hydras showed an increased mortality risk of approximately 25% (from 15% to 40%) when they were produced during weeks 5–9 corresponding for parents to the pathological phase, compared with weeks 0–4 where tumours are still unnoticeable (figure 6; GLMM, OR = 8.10, s.e. = 5.88, *p*‐value = 0.004). For buds issued from tumour-free hydras and TFTP, the mortality risk remained the same between the two phases.

The analysis of tumour transmission rate was conducted only on buds from tumoural hydras as no tumours were observed on buds from tumour-free control hydras. Thus, the model predicting the transmission rate in the MT40 tumoural strain included the parental status and the phase as fixed effects and controlled for the parental identity as a random effect. The chances of developing tumours are greatly reduced when the bud is derived from TFTP (figure 6; GLMM, *effect of the parental status*, reference group: tumoural hydras, OR = 0.02, s.e. = 0.02, *p*‐value = 0.001). On the other hand, when the bud was produced by a parent which subsequently developed a tumour, its probability of becoming tumoural was approximately four times higher. This is also the case if it is produced during the pathological phase of the parent, i.e. when the parent has already developed a tumour (figure 6; GLMM, *effect of the phase of bud production*, reference group: weeks 5–9, OR = 0.36, s.e. = 0.19, *p*‐value = 0.049). Regarding survival time, tumoural hydras, tumour-free hydras and TFTP all have a half-life of approximately 100 days, as no effect was selected during model selection.

### (c) Cell type and bacteriome

Concerning the cell type composition presented in table 1, tumoural hydras showed a 10-fold higher ratio of large interstitial stem cells to epithelial cells (LISC/EC) compared with TFTP and tumour-free hydras. In addition, their large to small interstitial stem cells (LISC/SISC) ratio was 20 times higher than that observed in the other two statuses. Thus, as the ratio of small interstitial stem cells to epithelial cells (SISC/EC) was similar for all three statuses, the tumours seemed to result from overproliferation of large interstitial stem cells.

**Table 1.** Ratios of large interstitial stem cells to epithelial cells (LISC/EC), small interstitial stem cells to epithelial cells (SISC/EC), and large interstitial stem cells to small interstitial stem cells (LISC/SISC) according to the status.

status	ratios		
	LISC/EC	SISC/EC	LISC/SISC
tumoural	2.25	0.63	3.56
TFTP	0.15	0.87	0.18
tumour-free	0.25	1.39	0.18

**Figure 7.**
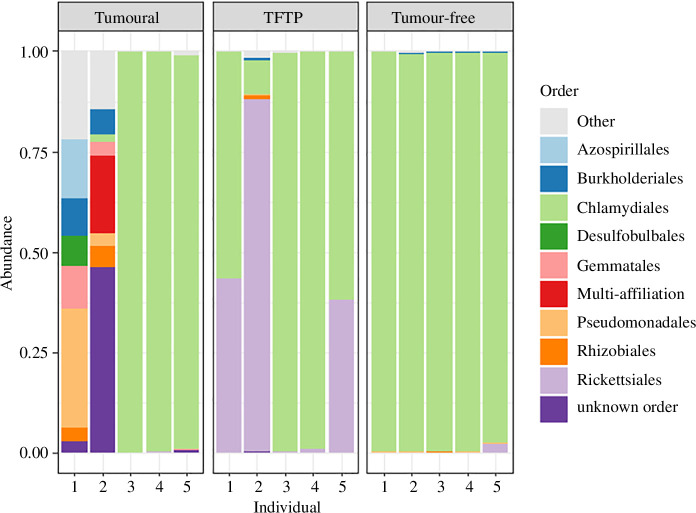
Abundance of the 10 most represented bacterial orders in the bacteriome of each individual analysed in each status.

Regarding the abundance of the 10 most represented orders of bacteria in tumoural hydras, tumour-free and TFTP, they all appear to be mainly colonized by Chlamydiales (see figure 7). Bacterial microbiota of polyps differed from that of their surrounding environment (i.e. water) not matter the alpha-diversity indices used (observed *F* value = 119.7, *p*‐value = 0.001; Chao1 *F* value = 82.34, *p*‐value = 0.001; Shannon *F* value = 32.45, *p*‐value = 0.001; invSimpson *F* value = 7.068, *p*‐value = 0.01) and did not differentiate with respect to polyp status (electronic supplementary material, figure S1), indicating that bacterial taxa identified on polyps probably represent taxa genuinely associated with the polyps and not contaminants from the environment. Alpha diversity did not differ significantly between the three groups (Kruskal–Wallis χ^2^ = 1.82, *p* = 0.403; electronic supplementary material, figure S2). However, the Adonis analyses showed that the bacteriome composition of the three groups differed from each other significantly or marginally significantly (Bray–Curtis index: *F* = 1.97, *p* = 0.054, electronic supplementary material, figure S3; Jaccard presence–absence index: *F* = 1.536, *p* = 0.002, electronic supplementary material, figure S4). Nevertheless, in both cases, it was the tumour-free group that differed from the other two (tumour-free versus TFTP, Bray–Curtis index, adjusted *p* = 0.040; Jaccard presence–absence index, adjusted *p* = 0.030; tumour-free versus tumoural, Bray–Curtis index, adjusted *p* = 0.040; Jaccard presence–absence index, adjusted *p* = 0.080), while tumoural and TFTP showed no significant differences (tumoural versus TFTP, Bray–Curtis index, adjusted *p* = 0.310; Jaccard presence–absence index, adjusted *p* = 0.120). The principal coordinate analyses of the Bray–Curtis index and Jaccard index according to the status are available in electronic supplementary material, figure S1, along with a graphical representation of alpha diversity for each status.

## 4. Discussion

As transmissible cancers are most often detected once they have already spread within host populations, the conditions associated with their emergence remain poorly understood. The cnidarian *H. oligactis* is emerging as a particularly promising model to investigate the evolution of transmissible tumours because it can develop spontaneous tumours relatively easily in the laboratory [26], and one laboratory strain harbours a transmissible tumour [24]. In this experimental study, we succeeded for the first time to observe the evolution of a transmissible tumour in the hydra. We have also shown that the rate of transmission of spontaneous tumours can increase over time; namely, four times in just five generations of selection. Finally, it is remarkable to note that this fifth generation of hydras carrying transmissible tumours is already showing changes in its life-history traits compared with its healthy counterparts.

First, this study confirms that *H. oligactis* is a species that easily develops spontaneous tumours in the laboratory [26]. In line with the tumours described by Boutry *et al.* [26], those from this study (that are coming from hydras collected in the same sampling area) also involve abnormal proliferation of large interstitial stem cells. However, in contrast to the tumours described by Rathje *et al.* [25], these tumours do not appear to be associated with a specific microbiota (since the tumoural and tumour-free individuals from the same strain did not differ from each other in microbiota composition). The reason why *H. oligactis* of the St Petersburg strain, unlike these hydras or those of the *Pelmatohydra robusta* strain (see [25]), diverge in their reliance on a particular bacteriome for tumour initiation and maintenance needs to be elucidated by further studies.

Our findings regarding the occurrence of tumours in the descendants of individuals with tumours are prima facie consistent with three potential scenarios. One possibility is that these tumours are also spontaneous (i.e. not vertically transmitted from their parents). A second hypothesis suggests parental hydras transmit to their offspring a genetic vulnerability that predisposes them to tumour formation under some conditions. The final possibility is that the tumour itself is transmitted. We favour this last scenario for several reasons. Firstly, the observation that the rate of tumour development in descendants of hydras with tumours is higher than the one of tumour-free hydras reared under the same conditions, implies that these tumours are not spontaneous. In addition, the fact this rate increases over time reinforces this statement. Secondly, the fact that the offspring of TFTP, which share the same genetics as the tumour-bearing hydras but remain tumour-free, do not develop as many tumours as the offspring of tumoural parents, indicates that we cannot attribute the issue to a genetic predisposition for tumour development. Therefore, the most likely explanation for these results is that the tumours observed in the offspring of tumoural parents are transmissible, like those observed in the St Petersburg strain.

Concerning the transmissibility, our findings remarkably indicated that some spontaneous tumours can be immediately transmissible, even if this was not systematic. However, since we lack information on whether the wild individuals used to establish the different strains had different genotypes or were clones when sampled, we cannot draw definitive conclusions regarding the precise proportion of spontaneous tumours that are directly transmissible. To assess the risk of pseudo-replication in this context, further studies would be necessary to estimate the number of genetically distinct hydras in field samples, as well as the probability of two genetically identical hydras both developing spontaneous, transmissible tumours. In addition to being sometimes immediate, tumour transmissibility can be artificially selected across generations. It is important to highlight that if an individual did not develop a tumour within two months, the follow-up observations were terminated. This interruption occurred without knowing whether tumours would later develop or if the individuals had the potential to produce buds that could develop tumours. In both scenarios, this could result in an underestimation of the transmission rate. Despite the fact that tumours have acquired the ability to be transmitted, this ability appears to be fragile, as it is particularly vulnerable to environmental conditions such as the availability of food. For instance, we observed a reduction in the rate of tumour transmission from 90% to 50% in one generation between the end of the experimental selection of tumour transmissibility (fifth generation) and the start of the analysis of life-history traits. This change coincides with a modification in the feeding protocols between the two experiments, leading to reduced food availability during the measurement of life-history traits. This observation is consistent with a previous study [27] that demonstrated a close link between diet and tumoural development in *H. oligactis*. The propensity to develop tumours, as well as the rate at which they develop, is positively correlated with the amount of food. Thus, this study suggests that the ability of spontaneous tumours that acquire transmissibility to maintain it, is also dependent on food intake.

In contrast to the first identified transmissible tumour in hydra (St Petersburg strain), which exhibits a constant transmission rate (71% at two weeks when asymptomatic and 71% at five weeks when symptomatic) [29], our current study on the MT40 strain reveals that the tumour is twice as likely to be transmitted when the host has entered the pathological phase (i.e. developed a tumour) compared with the pre-pathological phase (25% at two weeks versus 50% from the fifth week). Several non-exclusive scenarios may account for this disparity. Firstly, during the tumour transmission selection protocol, only buds born during the pathological phase of their parents were surveyed, potentially biasing the selection towards transmission during the pathological phase and resulting in a lower transmission rate in the pre-pathological phase. A second explanation is that the tumours from the St Petersburg strain had more time (i.e. 15 years in mass culture) to optimize their transmission, thereby improving it even during the early stages of the tumorigenesis, i.e. during the pre-pathological stage. This hypothesis would further support the conclusion that transmissibility is a selectable trait. Another explanation could be attributed to the distinct aetiology of the tumour. In the St Petersburg strain, tumour development is induced and persists only in the presence of a specific bacteriome [25], absent in the MT40 strain. Our results could be explained if we assume that bacteriome transmission remains constant regardless of the age of the hydra, leading to similar tumour development thereafter. However, when tumour development depends on the initial tumour cells’ number transmitted rather than the bacteriome (i.e. MT40), it is expected that symptomatic individuals could be more likely to infect their offspring than those in the pre-pathological stage.

Regarding life-history traits, the tumoural hydra of the MT40 strain exhibits, compared with a tumour-free population, (i) a similar first bud date, (ii) an initial increase in asexual reproductive effort only preceding tumour development, followed by a substantial decrease in budding rate thereafter, (iii) higher mortality in buds produced after the appearance of tumours, and (iv) a comparable survival time. The tumour appears to exert a detrimental influence on hydra budding immediately after the development of external tumoural manifestations, without diminishing overall survival. This could suggest a castration-like phenomenon akin to certain host–parasite systems [41], ultimately leading to a decline in fitness. In conjunction with the lower transmissibility of the tumour in the pre-pathological phase (i.e. before tumour appearance), these modifications suggest an adjustment of life-history traits of the host to offset the tumour’s costs by producing more buds when they are more likely to survive and remain tumour-free. Interestingly, Boutry *et al*. [29] also found that tumoural hydras from the St Petersburg strain adjust their life-history traits. However, what is remarkable here is that such adjustments can appear from the fifth generation, i.e. in only a few months. Further research is needed to specify when hydras bearing transmissible tumours start to adjust their life-history traits. Similarly, additional experiments are necessary to clarify the mechanisms responsible for these alterations (e.g. gene frequency changes, epigenetic modifications and phenotypic adjustments).

This study has enabled the evaluation, for the first time, of transgenerational effects of tumours on their host by investigating life-history traits in TFTP. These polyps have similar characteristics to those of tumoural polyps during the pre-pathological phase, but their asexual reproduction rate does not diminish after the fifth week as in tumoural polyps. Consequently, it seems that hydras that do not inherit tumours from their tumoural parents still inherit some tumoural factors that induce an early increase in budding, and this increase is remarkably not subsequently hindered by the presence of tumours. This result, for the initial nine weeks, in a superior asexual fitness compared with both tumoural and tumour-free polyps. Although we have not been able to measure trade-offs in TFPT, we cannot rule out that more in-depth studies would detect them. This could manifest as a reduction in budding rates occurring later (beyond nine weeks) or modifications in sensitivity to biotic and abiotic stress, for instance. Therefore, further research is necessary to comprehensively evaluate the transgenerational effects of tumours on overall fitness.

In the Perfect Storm theory initially proposed by Ujvari *et al*. [15] and further developed by Tissot *et al*. [16], two critical barriers have been proposed to explain the evolution of transmissible cancers. The first barrier pertains to the acquisition of transmissibility by tumour cells, while the second relates to the capacity to spread in host populations. The results of the present study suggest, at least in a hydra model, that the first barrier does not appear to be a major obstacle since a significant proportion of spontaneous tumours are immediately transmissible. Indirectly, these results support the idea that the scarcity of transmissible cancers in ecosystems is probably more attributed to the lack of suitable ecological conditions for their spread within populations, i.e. the second barrier (e.g. the presence of predators that would rapidly eliminate diseased individuals, the sensitivity of tumours to environmental stress—such as food availability, accumulation of deleterious mutations). If this conclusion is confirmed in the future, it is crucial to consider these aspects in the study of ecosystems disturbed by human activities, as they could potentially modify the conditions that favour the spread of transmissible cancers (see [42]).

Finally, it is important to highlight the significant distinction between this vertical transmission experiment and vertical transmission in nature. In nature, vertical transmission should disfavour the propagation of cancer cells (or any other parasite). A cancer cell lineage that is transmitted only vertically could not be maintained over time if transmission were solely vertical because the affected lineages would have lower fitness than unaffected lineages. Our experimental procedure has actively enhanced the fitness of vertically transmitted tumours by positively selecting host lineages that have the tumour. Indirectly, this suggests that the presence of transmissible tumours in wild hydra would imply some horizontal transmission of the tumour cells or a pathogen that could be contributing to tumorigenesis. However, until now, no tumorous hydras have been directly found in the field (despite many investigations), in accordance with the hypothesis that transmission occurs only vertically in this system.

## Data Availability

Scripts and data associated are provided in supplementary information [43]. The 16S rRNA raw sequence files for this study have been deposited in FASTQ format and can be found in the Sequence Read Archive from NCBI (BioProject: PRJNA1072327) [44].
